# Renewing focus on family planning service quality globally

**DOI:** 10.1186/s40834-016-0021-6

**Published:** 2016-06-22

**Authors:** Nancy L. Hancock, Gretchen S. Stuart, Jennifer H. Tang, Carla J. Chibwesha, Jeffrey S. A. Stringer, Benjamin H. Chi

**Affiliations:** 1 Department of Obstetrics and Gynecology, University of North Carolina School of Medicine, Campus Box 7577, Chapel Hill, NC 27599-7577 USA; 2 Centre for Infectious Disease Research in Zambia, PO Box 34681, 5032 Great North Road, Lusaka, Zambia; 3 UNC Project-Malawi, Tidziwe Centre, Private Bag A-104, Lilongwe, Malawi

**Keywords:** Family planning, Contraception, Quality, Global health

## Abstract

Reducing the global unmet need for contraception is currently a priority for many governments, multi-lateral initiatives, non-governmental organizations, and donors. Evidence strongly suggests that the provision of quality family planning services can increase uptake, prevalence, and continuation of contraception. While an accepted framework to define the components of family planning service quality exists, translating this framework into assessment tools that are accessible, easily utilized, and valid for service providers has remained a challenge. We propose new approaches to improve the standardization and accessibility of family planning service quality assessment tools to simplify family planning service quality evaluation. With easier approaches to program evaluation, quality improvements can be performed more swiftly to help increase uptake and continuation of contraception to improve the health of women and their families.

## Introduction

Each year, as many as 220 million women worldwide have an unmet need for contraception [1]. As a consequence, 40 % of the 210 million annual pregnancies are unintended [2]. Effective and accessible family planning (FP) services can bridge this important gap, with public health benefits that extend beyond the prevention of pregnancy alone: averted maternal morbidity and mortality, including from unsafe abortion; diminished infant morbidity and mortality via increased inter-pregnancy intervals and delayed first birth; and a lower burden of pediatric AIDS in high HIV prevalence settings [3]. Contraceptive use has also been associated with higher income via paid employment, increased access to education, and improved environmental sustainability, including greater access to sanitation, water, and food [4, 5]. Global efforts to expand FP services thus have great potential to save and improve lives, and contribute broadly to the Sustainable Development Goals [6, 7].

In response to the unmet need for FP services, especially in less developed countries, new efforts have systematically identified and addressed barriers to access, introducing context-specific, evidence-based strategies to increase FP utilization [3, 8]. Such efforts include checklists, text message interventions, and integrated services following delivery or alongside childhood immunizations [9–11]. Parallel efforts are needed to evaluate—and improve where necessary—healthcare quality in the area of FP. This critical point was emphasized by Bruce nearly 25 years ago [12]: “Improvements in the quality of services will result in a larger, more committed clientele of satisfied contraceptive users. Over the long term, this expanded base of well-served individuals will translate into higher contraceptive prevalence and, ultimately, reductions in fertility.”

Recent recommendations have once again brought the issue of program quality back to the forefront of FP policy and programs [13]. Quality is a cornerstone of the World Health Organization’s rights-based approach to FP [14] and the U.S. Centers for Disease Control and Prevention’s reproductive life planning [15] and provision of FP services [16]. Despite renewed interest, recent published data assessing FP quality are limited, especially in resource-constrained settings where unmet need may be greatest. Governments, donor organizations, and implementing partners such as Population Science International, Jhpiego, and Marie Stopes International monitor FP program quality [17]. However, the assessment methodologies are often not standardized, making these results difficult to interpret and compare. In addition, tools for evaluating programs vary in structure and content and can be poorly accessible to frontline providers and managers. In this paper, we review methods for measuring quality of FP services and propose several broad approaches to help maximize the benefits of FP quality assessments globally.

## Quality of family planning services influences use

The association between quality of FP services and the uptake and continuation of contraception has been demonstrated in numerous settings. The classic study that demonstrated this relationship was conducted in Bangladesh from 1989 to 1991. Repeated household surveys were conducted among 3,497 women over 30 months to assess care provided by female fieldworkers. Non-contraceptive users at baseline who deemed their care to be of high quality were 27 % more likely to initiate a method and 41 % more likely to continue use compared to those who perceived low quality services (*p*-value < 0.05 for all reported comparisons). Contraceptive users at baseline who rated their care as high quality were 72 % more likely to continue use. The positive impact of quality on FP initiation and continuation persisted after controlling for the effects of programmatic and client-characteristic variables [18].

High quality FP clinics have also been associated with increased contraception continuation in the Philippines [19]. In this study, 1,728 new contraceptive users from 80 service delivery points were interviewed within 6 months of their initial visit to an FP clinic and again 18 months later. Women who rated their care as high quality were more likely to have continued their contraceptive method use during the study period compared to women who rated their care as low quality (65 % versus 53 %). This association persisted after controlling for fertility intentions and sociodemographic factors (*p*-value < 0.01). Similarly, in Peru, contraceptive use was nearly 2.5 times higher among women reporting high quality care compared to low (43 % versus 18 %) [20].

More recently, an analysis from Kenya linked a woman’s direct experience at the facility where she obtained family planning services to contraceptive use [21]. The analysis included 3,246 women and 260 facilities (87 public and 173 private). Providing a broader, consistent method mix; help with method selection; and being treated ‘very well’ increased the probability of contraceptive use (prevalence ratio 1.1). The relationship between being treated well and contraceptive use was strongest among younger, less educated women (prevalence ratio 1.4).

## Defining and measuring quality in family planning

An evidence-based framework to define quality of care specifically for FP has been established by Bruce [12] and comprises: choice of methods, information given to users, technical competence, interpersonal relations, follow-up or continuity mechanisms, and appropriate constellation of services (Table 1).

**Table 1:** Elements of Bruce’s evidence-based quality of family planning care framework

Element	Definition	Potential impact
Choice of methods	Number of available contraceptive methods	• Increased uptake of contraception [22, 23] • Increased continuation due to method flexibility allowing switching instead of stopping altogether [42] • Selected method that meets client’s specific needs [43, 44] • Increased likelihood that at least one method will be available, especially in settings with frequent stock-outs
Information given to users	Knowledge conveyed about available contraceptive methods including how to use, benefits and risks, and potential side effects	• Increased uptake of contraception due to dispelled myths and misconceptions [45] • Increased continuation rates due to recognition and management of side effects [46]
Technical competence	Correct and consistent application of medical eligibility criteria and routinely completing procedures to a defined standard	• Reduced risk of side effects and complications due to appropriate application of the WHO Medical Eligibility Criteria • Reduced risk of infection and improper placement of subdermal implants and intra-uterine devices [47]
Interpersonal relations	Treating clients with honesty, sympathy and understanding	• Increased uptake and continuation due to being treated with dignity and respect [48–50]
Follow-up or continuity mechanisms	Establishing when and how clients will return to clinic	• Decreased fertility rates due to increased contraceptive continuation rates [51]
Appropriate constellation of services	Making contraception readily available to clients regardless of where they access care	• Increased access to contraception via service integration, mobile delivery of services, and task-shifting

Choice of contraceptive methods is the essential underpinning of quality service provision. Having multiple contraceptive methods available – and also being able to choose from those methods—increases contraceptive uptake and continuation [22–24]. Information and counseling given to clients helps them understand the choices available to them; the advantages and disadvantages of different methods; and the recognition and management of side effects [25, 26]. Technical competence influences the rate of procedural complications and perceived discomfort at the time of contraceptive placement. It can also reduce health provider misconceptions that may unnecessarily raise barriers to service provision – such as requiring a woman to be menstruating in order to receive FP or a pelvic exam prior to initiating birth control pills [27]. Interpersonal relations affect the client’s perceptions of method efficacy, service satisfaction, and likelihood of return to the clinic.

The final domain in Bruce’s framework is the “appropriate constellation of services,” which is the most subjective and contextual of the elements. Health services for women are often segmented or inconsistent. FP counseling may not be provided in antenatal clinic, or post-partum lactating women who present for FP may be denied because they are not menstruating. Providers may also counsel patients differently depending on their age, number of prior pregnancies, and timing of recent pregnancies. Quality programs bridge these gaps by meeting the needs of women regardless of where they access care or when in their reproductive lives they seek care. The goal is to provide FP in a way that can respond to clients’ health needs, instead of imposing a demarcated and rigid health service delivery system. Provision of depot medroxyprogesterone via community health workers is one such example of how FP services can be more accessible and flexible [28].

Methods for measuring FP quality of care have generally been built upon the six part framework initially outlined by Bruce and have demonstrated correlations between FP clients’ reported satisfaction and presence of the Bruce framework [18–20, 25]. The Situation Analysis [29], Service Provision Assessment [30], and Quick Investigation of Quality [31] (Table 2) are standardized quantitative tools using the Bruce framework elements. These quantitative assessments confirm that the presence of the Bruce framework elements correlate with high quality FP services [32]. Such tools are intended to be used as part of a cycle for assessing, intervening, and evaluating for improvement. Collectively, these methodologies have been used over the last three decades in more than 50 countries worldwide to document FP program quality.

**Table 2:** Three established methodologies for measuring the quality of family planning (FP) services

Method	Details	Limitations
Situation Analysis [29]	• Designed specifically for FP • Facility inventory to assess the available services and physical structure, including types and amount of stock • FP provider interviews to determine the level of preparation based on training, experience, and degree of supervision • Client-provider observation to review service delivery and technical skills of providers • Client exit interviews to gather visit information from the client’s perspective	• Expensive • Time intensive • Training required to standardize observations and exit interviews • Difficult to repeat frequently
Service Provision Assessment [30]	• Designed for reproductive and child health, including FP • Same components as Situation Analysis • Different data collection tools	• Expensive • Time intensive • Training required to standardize observations and exit interviews • Difficult to repeat frequently
Quick Investigation of Quality [31]	• Derived from the Service Provision Assessment specifically for FP • Designed to be an efficient, low-cost, reproducible method to measure quality • Fewer components – no provider interview • Different data collection tools with fewer questions	• Training required to standardize observations and exit interviews

However, these FP quality assessments are typically performed on a national scale, which requires significant time and resources. Technical expertise to identify an appropriate sampling frame and perform analyses on the collected data is needed, which may not be readily available. Results are often aggregated; as a result, provinces, districts, and facilities may not receive individual data. Identifying a mechanism to provide the data to the facilities is ideal. However, for many facilities, data are not available either because the facility was not included or a limited number of clients were interviewed due to the sampling frame. In addition, because these methods rely on interactions between study staff, clinic staff, and clients, the data may not be reliable or valid due to courtesy bias, recall bias, and lack of response validation. Finally, none identify why FP services are not used as each depends solely on respondents utilizing FP services. Changes to improve the quality of FP services can be difficult to make because of these challenges associated with surveillance-oriented assessments. Primary care initiatives such as SafeCare are attempting to address these shortcomings through structured and validated processes tailored to different levels of healthcare in international settings [33]. We advocate a similar approach but targeted specifically to FP service quality.

## Towards a universal framework to improve family planning quality

We describe seven key approaches to maximize FP quality assessment. First, site-level tools for regular quality assessment need to be developed, adapted, and easily available for a variety of settings. A few such FP quality tools are readily available, such as the SEED Assessment Guide from EngenderHealth, a community FP sustainability checklist, and resources cited by the US Family Planning National Training Centers [34–36]. These instruments—and others like them—should be reviewed and a set of consensus core elements agreed upon to define FP quality across a variety of service provision models (e.g., health facilities, community outreach, mobile units). Adapted tools incorporating these core elements should be validated and made publically available.

Second, in addition to ongoing facility-based evaluations, regular community-level assessments are needed to determine the FP needs of—and barriers to access among—persons who do not regularly engage in care. Community-level assessments may not provide information about service quality directly, but they can reveal important insights, including community myths and misconceptions that may be difficult to obtain in the facility setting. Such information could directly inform local strategies to improve uptake and optimize FP services, which in turn can be monitored by ongoing facility-based assessments. Efforts are needed to catalogue community-assessment tools from governments, organizations, and donors.

Third, standardized FP quality indicators and methodologies for data analyses are needed to help identify gaps in service provision as well as potential points for intervention. For example, to appropriately counsel patients on available options, one site may need more assistance with commodity procurement while another needs additional training. Standardization will also allow generation of program “report cards” to facilitate monitoring for improvements over time and comparisons between sites to help structure resource allocation. Such standardization will also allow comparisons between different program formats, such as outreach clinic days, mobile services, public clinics, and franchised clinics, to help determine which approaches should be scaled-up due to high efficacy.

Fourth, continuous quality improvement (QI) techniques can be used to further explore barriers, design strategies, and monitor incremental progress. Recent examples using root cause analysis and Plan-Do-Study-Act cycles, two established QI methods, increased FP uptake and continuation in Afghanistan, Egypt, and Tunisia [37–39]. In addition, using a phased roll-out approach for changes can assist in the refinement and optimization of promising interventions at the facility, prior to widespread scale-up.

Fifth, given the potential for structural barriers to healthcare improvement, early engagement of local health authorities—as well as local policy makers—is critical to the success of such endeavors. Government officials may need to authorize proposed changes to FP service provision, especially when trying to expand access to long-acting reversible contraception for minors. Similarly, health authorities may need to authorize task shifting activities that increase access to FP, such as allowing mid-level providers to perform sterilizations.

Sixth, new and innovative responses to FP quality assessments must be implemented and rigorously evaluated. One area of great promise, for example, is performance-based incentive (PBI) programs. PBI programs enhance intrinsic motivation by rewarding FP providers for providing quality services. PBI programs are especially relevant in settings where health care provider motivation to improve services is low because of insufficient staffing or compensation or because improving quality may increase workload. Such initiatives have been implemented in Kenya, Burundi, and Liberia with early signs of success and have included the use of quarterly quality of care checklists and patient satisfaction surveys to determine how much a provider and/or the facility will receive as compensation for reaching predetermined target scores for quality care [40]. However, such services must be carefully designed to ensure appropriateness for local context and setting of reasonable targets. It is also critical to ensure that PBI programs do not result in patient coercion (or appearance of coercion) and do not act to incentivize providers to provide FP services at the expense of other essential services, such as HIV or maternity care.

Finally, we encourage multidisciplinary approaches that consider the range of clinical and public health expertise in development of a more robust evaluation framework. Providers and program implementers need to be engaged with researchers to help ensure the implementation of quality FP services at individual, regional, and health system levels. As stated by Mamoud Fathalla, “The question should not be why do women not accept the service we offer, but why do we not offer a service that women will accept” [41].

## Conclusions

Quality in FP service provision has been long recognized as a central component to increase contraceptive uptake, prevalence, and continuation. Despite renewed interest in this area—from governments, donor agencies, and multilateral initiatives such as FP2020—there often exists a tenuous, and at times interrupted, link between FP quality assessment and services provided. Focused and frequent quality assessments will provide actionable data to program managers. Identifying, and then acting upon, systematic barriers to low FP service quality will decrease the unmet need for contraception and lead to important gains in women’s health.

## Abbreviations

FP, family planning; PBI, performance based incentives; QI, quality improvement.

## References

[1] Singh S, Darroch JE, Singh S, Darroch JE (2012). Adding it up: Costs and benefits of contraceptive services - Estimates for 2012.

[2] Singh S, Sedgh G, Hussain R (2010). Unintended Pregnancy: Worldwide Levels, Trends, and Outcomes. Stud Fam Plann.

[3] Greene M, Joshi S, Robles O (2012). By choice, not by chance: Family planning, human rights, and development.

[4] Cleland J, Bernstein S, Ezeh A, Faundes A, Glasier A, Innis J (2006). Family planning: the unfinished agenda. Lancet.

[5] Canning D, Schultz TP (2012). The economic consequences of reproductive health and family planning. Lancet.

[6] Petruney T, Wilson LC, Stanback J, Cates W (2014). Family planning and the post-2015 development agenda. Bull World Health Organ.

[7] Stenberg K, Axelson H, Sheehan P, Anderson I, Gülmezoglu AM, Temmerman M, Mason E, Friedman HS, Bhutta ZA, Lawn JE, et al. Advancing social and economic development by investing in women's and children's health: a new Global Investment Framework. The Lancet. 2014;383:1333–54. 10.1016/S0140-6736(13)62231-X 24263249

[8] Jacobstein R, Curtis C, Spieler J, Radloff S (2013). Meeting the need for modern contraception: effective solutions to a pressing global challenge. Int J Gynaecol Obstet.

[9] FHI 360 (2013). PROGRESS end-of-project report: Meeting the family planning needs of underserved populations.

[10] Stang A, Schwingl P, Rivera R (2000). New contraceptive eligibility checklists for provision of combined oral contraceptives and depot-medroxyprogesterone acetate in community-based programmes. Bull World Health Organ.

[11] Halpern V, Lopez LM, Grimes DA, Stockton LL, Gallo MF (2013). Strategies to improve adherence and acceptability of hormonal methods of contraception. Cochrane Database Syst Rev.

[12] Bruce J (1990). Fundamental elements of the quality of care: a simple framework. Stud Fam Plan.

[13] World Health Organization (2006). Quality of care: A process for making strategic choices in health systems.

[14] World Health Organization (2014). Ensuring human rights in the provision of contraceptive information and services: Guidance and recommendations.

[15] Johnson K, Posner SF, Biermann J, Cordero JF, Atrash HK, Parker CS, Boulet S, Curtis MG (2006). Recommendations to improve preconception health and health care--United States. A report of the CDC/ATSDR Preconception Care Work Group and the Select Panel on Preconception Care. MMWR Recomm Rep.

[16] Gavin L, Moskosky S, Carter M, Curtis K, Glass E, Godfrey E, Marcell A, Mautone-Smith N, Pazol K, Tepper N, Zapata L (2014). Providing quality family planning services: Recommendations of CDC and the U.S. Office of Population Affairs. MMWR Recomm Rep.

[17] Blumenthal PD, Shah NM, Jain K, Saunders A, Clemente C, Lucas B, Jafa K, Eber M (2013). Revitalizing long-acting reversible contraceptives in settings with high unmet need: a multicountry experience matching demand creation and service delivery. Contraception.

[18] Koenig MA, Hossain MB, Whittaker M (1997). The Influence of Quality of Care upon Contraceptive Use in Rural Bangladesh. Stud Fam Plan.

[19] RamaRao S, Lacuesta M, Costello M, Pangolibay B, Jones H (2003). The link between quality of care and contraceptive use. Int Fam Plan Perspect.

[20] Mensch B, Arends-Kuenning M, Jain A (1996). The Impact of the Quality of Family Planning Services on Contraceptive Use in Peru. Stud Fam Plan.

[21] Tumlinson K, Pence BW, Curtis SL, Marshall SW, Speizer IS (2015). Quality of Care and Contraceptive Use in Urban Kenya. Int Perspect Sex Reprod Health.

[22] Kols AJ, Sherman JE. Family planning programs: improving quality. Popul Rep J 1998;47:1–39. 9926377

[23] Pariani S, Heer DM, Arsdol MDV (1991). Does Choice Make a Difference to Contraceptive Use? Evidence from East Java. Stud Fam Plan.

[24] Sedgh G, Hussain R (2014). Reasons for Contraceptive Nonuse among Women Having Unmet Need for Contraception in Developing Countries. Stud Fam Plann.

[25] Sanogo D, RamaRao S, Jones H, N'Diaye P, M'Bow B, Diop CB (2003). Improving quality of care and use of contraceptives in Senegal. Afr J Reprod Health.

[26] Kaufman J, Zhirong Z, Xinjian Q, Yang Z (1992). The Quality of Family Planning Services in Rural China. Stud Fam Plann.

[27] Tepper NK, Curtis KM, Steenland MW, Marchbanks PA (2013). Physical examination prior to initiating hormonal contraception: a systematic review. Contraception.

[28] Chin-Quee D, Bratt J, Malkin M, Nduna MM, Otterness C, Jumbe L, Mbewe RK (2013). Building on safety, feasibility, and acceptability: the impact and cost of community health worker provision of injectable contraception. Glob Health Sci Pract.

[29] Miller R, Fisher A, Miller K, Ndhlovu L, Maggwa BN, Askew I, Sanogo D, Tapsoba P (1997). The situation snalysis approach to assessing family planning and reproductive health services: A handbook.

[30] MEASURE Evaluation PRH. Service delivery - Quality of care/service provision assessment [http://www.cpc.unc.edu/measure/prh/rh_indicators/crosscutting/service-delivery-ii.h.2]. Accessed 9 July 2014 and 21 Mar 2016.

[31] MEASURE Evaluation, Monitoring and Evaluation Subcommittee of the Maximizing Access and Quality Initiative. Quick investigation of quality (QIQ): A user's guide for monitoring quality of care in family planning. In: MEASURE Evaluation Manual Series, vol. No 2. Chapel Hill: MEASURE Evaluation. Carolina Population Center, University of North Carolina at Chapel Hill; 2001.

[32] Sullivan T, Bertrand J (2000). Monitoring quality of care in family planning by the Quick Investigation of Quality (QIQ): Country reports. MEASURE Evaluation Technical Report Series No 5.

[33] SafeCare: Basic healthcare standards. [http://www.safe-care.org/]. Accessed 15 Mar 2015.

[34] EngenderHealth (2011). The SEED assessment guide for family planning programming.

[35] Quality family planning recommendations: Resources to support implementation (As of April 23, 2015). [http://fpntc.org/training-and-resources/quality-family-planning-recommendations-resources-to-support-implementation]. Accessed 16 Mar 2015 and 21 Mar 2016

[36] Arscott-Mills S, Foreman M, Graham V (2012). Family planning sustainability checklist: A project assessment tool for designing and monitoring sustainability of community-based family plannning services.

[37] Tawfik Y, Rahimzai M, Ahmadzai M, Clark PA, Kamgang E (2014). Integrating family planning into postpartum care through modern quality improvement: experience from Afghanistan. Glob Health Sci Pract.

[38] Letaief M, Ben Hmida A, Mouloud B, Essabbeh B, Ben Aissa R, Gueddana N (2008). Implementing a quality improvement programme in a family planning centre in Monastir, Tunisia. East Mediterr Health J.

[39] Hong R, Mishra V, Fronczak N (2011). Impact of a quality improvement programme on family planning services in Egypt. East Mediterr Health J.

[40] Morgan L (2012). Can incentives strengthen access to quality family planning services? Lessons from Burundi, Kenya, and Liberia.

[41] van den Broek NR, Graham WJ (2009). Quality of care for maternal and newborn health: the neglected agenda. BJOG.

[42] Phillips JF, Simmons R, Koenig MA, Chakraborty J (1988). Determinants of reproductive change in a traditional society: evidence from Matlab, Bangladesh. Stud Fam Plan.

[43] Bhatia S, Mosley WH, Faruque ASG, Chakraborty J (1980). The Matlab Family Planning-Health Services Project. Stud Fam Plan.

[44] Steele F, Curtis SL, Choe M (1999). The Impact of Family Planning Service Provision on Contraceptive-use Dynamics in Morocco. Stud Fam Plan.

[45] Henry-Lee A (2001). Women's reasons for discontinuing contraceptive use within 12 months: Jamaica. Reprod Health Matters.

[46] Cotten N, Stanback J, Maidouka H, Taylor-Thomas JT, Turk T (1992). Early Discontinuation of Contraceptive Use In Niger and The Gambia. Int Fam Plan Perspect.

[47] World Health Organization Department of Reproductive Health and Research (WHO/RHR), Johns Hopkins Bloomberg School of Public Health/Center for Communication Programs (CCP) Knowledge for Health Project (2011). Family planning: A global handbook for providers (2011 update).

[48] Kim Y-M, Rimon J, Winnard K, Corso C, Mako IV, Lawal S, Babalola S, Huntington D (1992). Improving the Quality of Service Delivery in Nigeria. Stud Fam Plann.

[49] Schuler SR, McIntosh EN, Goldstein MC, Pande BR (1985). Barriers to Effective Family Planning in Nepal. Stud Fam Plann.

[50] Vera H (1993). The Client's View of High-Quality Care in Santiago, Chile. Stud Fam Plann.

[51] Jain AK (1989). Fertility reduction and the quality of family planning services. Stud Fam Plan.

